# Effects of Environmental Enrichment on Leg Health and Locomotor Traits of Broiler Chickens Fed Antibiotic or Multi‐Strain Probiotic Performance Enhancers

**DOI:** 10.1111/asj.70233

**Published:** 2026-08-12

**Authors:** Lorraine Aline Barbosa de Lima, Caio Cesar dos Ouros, Maria Eliza Ribeiro da Silva, Elivelton de Salles da Silveira, Monaline Ribeiro Aquino da Silva, Maria Fernanda de Castro Burbarelli, Fabiana Ribeiro Caldara, Claudia Marie Komiyama, Irenilza de Alencar Nääs, Rodrigo Garófallo Garcia

**Affiliations:** ^1^ Universidade Federal da Grande Dourados Dourados Mato Grosso Brazil; ^2^ Programa de Pós‐Graduação em Engenharia de Produção Universidade Paulista (UNIP) São Paulo São Paulo Brazil

**Keywords:** additives, gait score, locomotor health, well‐being

## Abstract

Commercial broiler production faces the challenge of optimizing animal welfare and rapid genetic gains in growth rate, with locomotor disorders and footpad lesions remaining important limiting factors. Antibiotic growth promoters and probiotics are dietary strategies for modulating gut health, whereas environmental enrichment promotes physical development. This study evaluated the effects of dietary additives and environmental enrichment on welfare indicators in broiler chickens. A total of 1440 male chicks were assigned to a completely randomized 3 × 2 factorial design (control, antibiotic, and probiotic diets, with or without environmental enrichment), with eight replicates per treatment. Environmental enrichment consisted of a plastic ramp and rotating objects (mirrors, hay, chains, and balls). Locomotor traits, including gait score, pododermatitis, angular deformities, and latency to lie, were assessed. A significant interaction between dietary additives and environmental enrichment was observed for femoral degeneration, with probiotic‐fed birds under enriched conditions showing the lowest degeneration scores. Probiotics also improved gait score at 36 days, whereas both probiotics and antibiotics reduced pododermatitis severity. No effects were detected on tibial dyschondroplasia, spondylolisthesis, latency to lie, angular deformities, dorsocranial myopathy, or bone strength. The combination of probiotics and environmental enrichment positively influenced selected locomotor welfare indicators, whereas both dietary additives reduced pododermatitis without affecting major skeletal disorders.

## 1 Introduction

Modern poultry production has increasingly adopted alternatives to antibiotic growth promoters in response to concerns over antimicrobial resistance and food safety, with probiotics, prebiotics, and phytobiotics emerging as promising options (Rafiq et al. 2022). Among them, probiotics have the potential to modulate the intestinal microbiota, improve digestibility, and strengthen the immune system, reducing enteric diseases and favoring productive performance (Markowiak and Śliżewska 2018).

Emerging evidence also suggests that probiotics may influence skeletal development through the gut–bone axis. By increasing the production of short‐chain fatty acids, enhancing mineral absorption (particularly calcium and phosphorus), and modulating inflammatory cytokines involved in bone remodeling, probiotics may contribute to improved bone mineralization and structural integrity (Zaiss et al. 2019).

In addition, diets that promote intestinal balance contribute to more consistent feces and drier litter, reducing the incidence of pododermatitis and stimulating greater activity in birds (Kumar et al. 2025).

Despite genetic advances, the rapid growth of broilers compromises locomotor integrity, increasing the occurrence of lameness and locomotion difficulties (Knowles et al. 2008). In this context, environmental enrichment (EE) is a strategy to promote welfare by stimulating natural behaviors through various physical and sensory stimuli (Jones 2001; Xu et al. 2022). Resources such as perches, mirrors, and manipulable materials may reduce injuries and improve productive efficiency (Ghani et al. 2025), as well as increase activity and reduce locomotor problems (Blatchford et al. 2012; Ohara et al. 2015). Changes in body conformation resulting from accelerated growth also affect locomotion, necessitating less efficient biomechanical adaptations (Corr et al. 2003).

Dietary additives primarily influence physiological processes through modulation of the gut microbiota and immune function, whereas EE stimulates physical activity and musculoskeletal development; combining these strategies may produce complementary or even synergistic effects on locomotor health and skeletal integrity. However, whether these interventions interact to improve leg health in broiler chickens remains poorly understood.

Although dietary additives and EE have been independently associated with improvements in health and welfare, information regarding their combined effects on locomotor development and skeletal integrity in broilers remains limited. Therefore, we hypothesized that combining dietary additives with EE would produce greater improvements in locomotor health and skeletal integrity than either strategy alone. The objective of this study was to evaluate the combined and individual effects of dietary additives, including an antibiotic growth promoter and a probiotic blend, and EE on bone quality, structural limb disorders, locomotor performance, and pododermatitis in broiler chickens.

## 2 Materials and Methods

### 2.1 Animals, Housing, and Experimental Design

All animal procedures were approved by the Ethics Committee on the Use of Animals of the Faculty of Agricultural Sciences of the Federal University of Grande Dourados, Dourados, MS, Brazil (protocol no. 38.2022/CEUA).

A total of 1440 1‐day‐old male Ross 308 (TM4) broiler chicks, vaccinated against Marek's disease at the hatchery, were used. Birds were randomly assigned to 48 floor pens (30 birds per pen) in an experimental poultry house (50 × 10 m) at the Federal University of Grande Dourados. The house was equipped with a negative‐pressure ventilation system, and each 4.5 m^2^ pen contained a pendulum drinker, a tubular feeder, and wood‐shavings litter. Brooding was provided by 250 W infrared lamps, one per pen. An intermittent lighting program was adopted in accordance with the Ross management guidelines, with a light intensity of 22 lx. Feed and water were provided ad libitum throughout the experimental period. Experimental diets (Table 1) were formulated for the pre‐starter, starter, grower, and finisher phases to meet the nutritional requirements recommended by Rostagno et al. (2017). All other management practices followed the recommendations of the Ross broiler management manual.

**TABLE 1:** Experimental diet used in the pre‐starter, starter, grower, and finisher phases of broiler chickens fed antibiotic or multi‐strain probiotic performance enhancers.

Ingredients	1–7 days	8–21 days	22–35 days	36–42 days
Corn	42.858	46.260	53.137	62.819
Soybean meal	46.218	42.749	35.592	27.675
Soybean oil	5.096	5.862	6.182	5.003
Dicalcium phosphate	2.331	2.132	1.821	1.471
Limestone	0.762	0.721	0.557	0.424
Salt	0.489	0.473	0.449	0.420
dl‐Methionine	0.351	0.327	0.286	0.228
Mineral^1^ premix	0.050	0.050	0.050	0.050
Vitamin^2^ premix	0.028	0.025	0.017	0.015
l‐Lysine HCl	0.105	0.224	0.178	0.217
l‐Threonine	0.060	0.058	0.064	0.058
Inert ingredient^3^	1.652	1.109	1.657	1.670
Total	100.000	100.000	100.000	100.000
Calculated composition				
Crude protein (%)	25.00	23.75	21.04	18.29
Metabolizable energy (kcal/kg)	3000	3100	3200	3250
Calcium (%)	1.029	0.911	0.758	0.606
Sodium (%)	0.905	0.210	0.201	0.191
Chlorine (%)	0.236	0.226	0.212	0.296
Available phosphorus (%)	0.431	0.382	0.324	0.259
Digestible lysine (%)	1.368	1.366	1.161	1.00
Digestible Met + Cys (%)	1.099	0.955	0.856	0.742
Digestible Met (%)	0.667	0.627	0.558	0.473
Digestible threonine (%)	0.905	0.852	0.763	0.661

^1^ Poultry mineral supplement: 30 mg Fe, 6.5 mg Cu, 60 mg Mn, 50 mg Zn, 1.25 mg I, and 0.25 mg Se per kilogram of diet.

^2^ For 280 g/t of feed: 17,769 IU vitamin A, 5385 IU vitamin D, 25.20 mg vitamin E, 4.33 mg vitamin K, 2.91 mg vitamin B1, 10.66 mg vitamin B2, 5.38 mg vitamin B6, 0.03 mg vitamin B12, 43.94 mg niacin, 11.89 mg pantothenic acid, 0.97 mg folic acid, and 0.06 mg biotin.For 250 g/t of feed: 15,866 IU vitamin A, 4808 IU vitamin D, 22.50 mg vitamin E, 3.87 mg vitamin K, 2.60 mg vitamin B1, 9.52 mg vitamin B2, 4.81 mg vitamin B6, 0.02 mg vitamin B12, 39.23 mg niacin, 10.62 mg pantothenic acid, 0.87 mg folic acid, and 0.06 mg biotin per kilogram of diet. 170 g/t of feed: 10,789 IU of vitamin A, 3269 IU of vitamin D, 15.30 mg of vitamin E, 2.63 mg of vitamin K, 1.77 mg of vitamin B1, 6.47 mg of vitamin B2, 3.27 mg of vitamin B6, 0.02 mg of vitamin B12, 26.68 mg of niacin, 7.22 mg of pantothenic acid, 0.59 mg of folic acid, and 0.04 mg of biotin per kilogram of diet.For 150 g/t of feed: 9519 IU of vitamin A, 2885 IU of vitamin D, 13.50 mg of vitamin E, 2.32 mg of vitamin K, 1.56 mg of vitamin B1, 5.71 mg of vitamin B2, 2.88 mg of vitamin B6, 0.01 mg of vitamin B12, 23.54 mg of niacin, 6.37 mg of pantothenic acid, 0.52 mg of folic acid, and 0.03 mg of biotin.

^3^ Variable portion: inert material replaced by the respective diet: AMC = 0.05% zinc bacitracin 1–35 days; PROB = 0.02% probiotic 1–42 days.

The experiment was conducted in a completely randomized design with a 3 × 2 factorial arrangement, consisting of three dietary treatments (negative control, antibiotic growth promoter, and probiotic) and two environmental conditions (with or without EE), resulting in six treatment combinations, each with eight replicates.

Zinc bacitracin was used as the antibiotic growth promoter at the manufacturer's recommended inclusion level (500 g/ton). The probiotic consisted of a commercial blend containing *Bacillus subtilis* (minimum 3.60 × 10^9^ CFU/g), *Bifidobacterium bifidum* (minimum 2.50 × 10^9^ CFU/g), *Enterococcus faecium* (minimum 2.60 × 10^9^ CFU/g), and *Lactobacillus acidophilus* (minimum 1.30 × 10^9^ CFU/g). The probiotic was included following the manufacturer's recommendations (200 g/ton).

**FIGURE 1:**
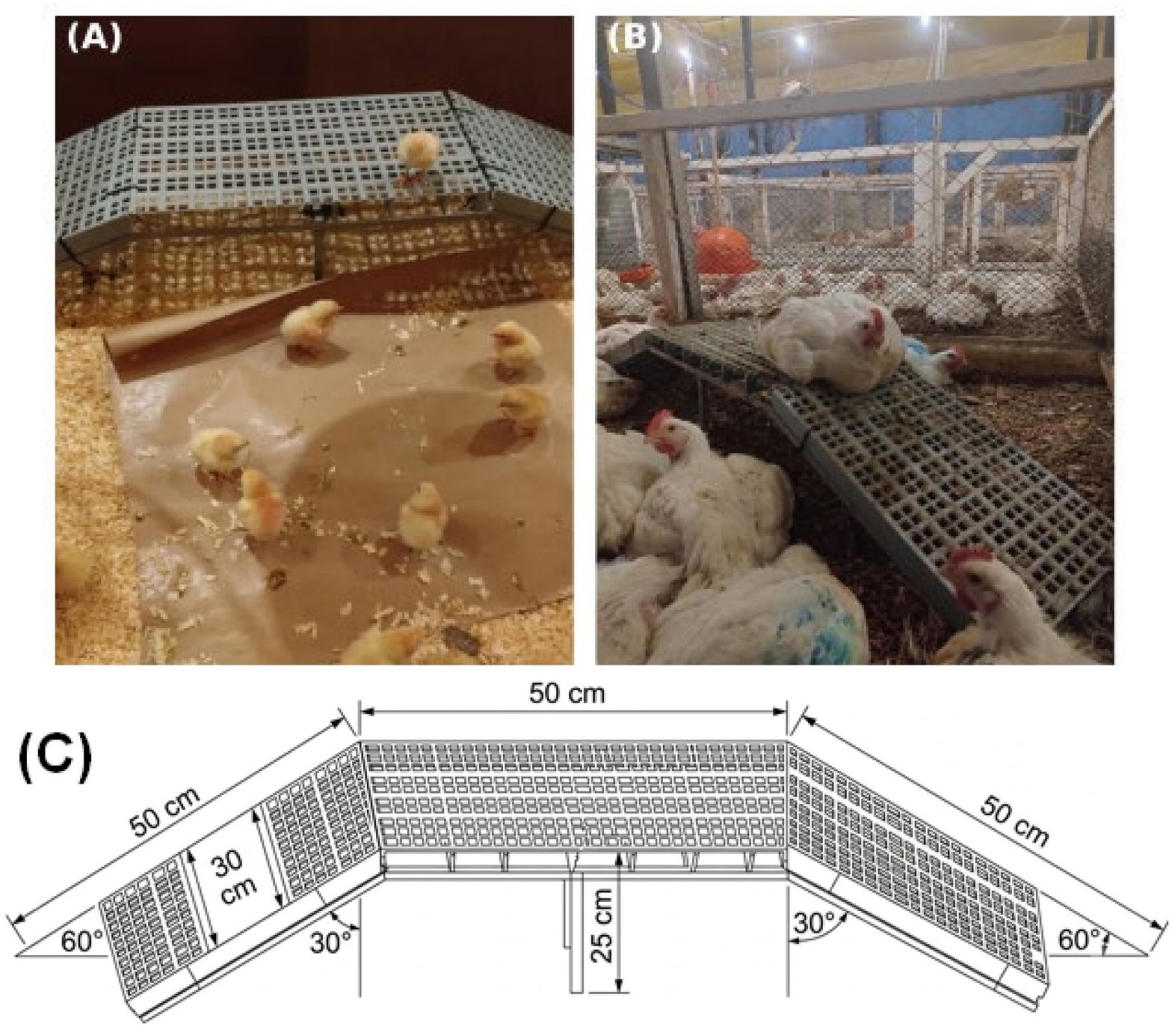
Permanent EE consisting of plastic ramps made of perforated flooring. (A) Young broilers using the enrichment structure and (B) older broilers using the same structure during the grow‐out period. (C) Plastic ramps scheme for better visualization of measurements. *Source:* Authors.

EE included both permanent and temporary components. The permanent enrichment consisted of a plastic ramp constructed from perforated plastic flooring (Figure 1).

**FIGURE 2:**
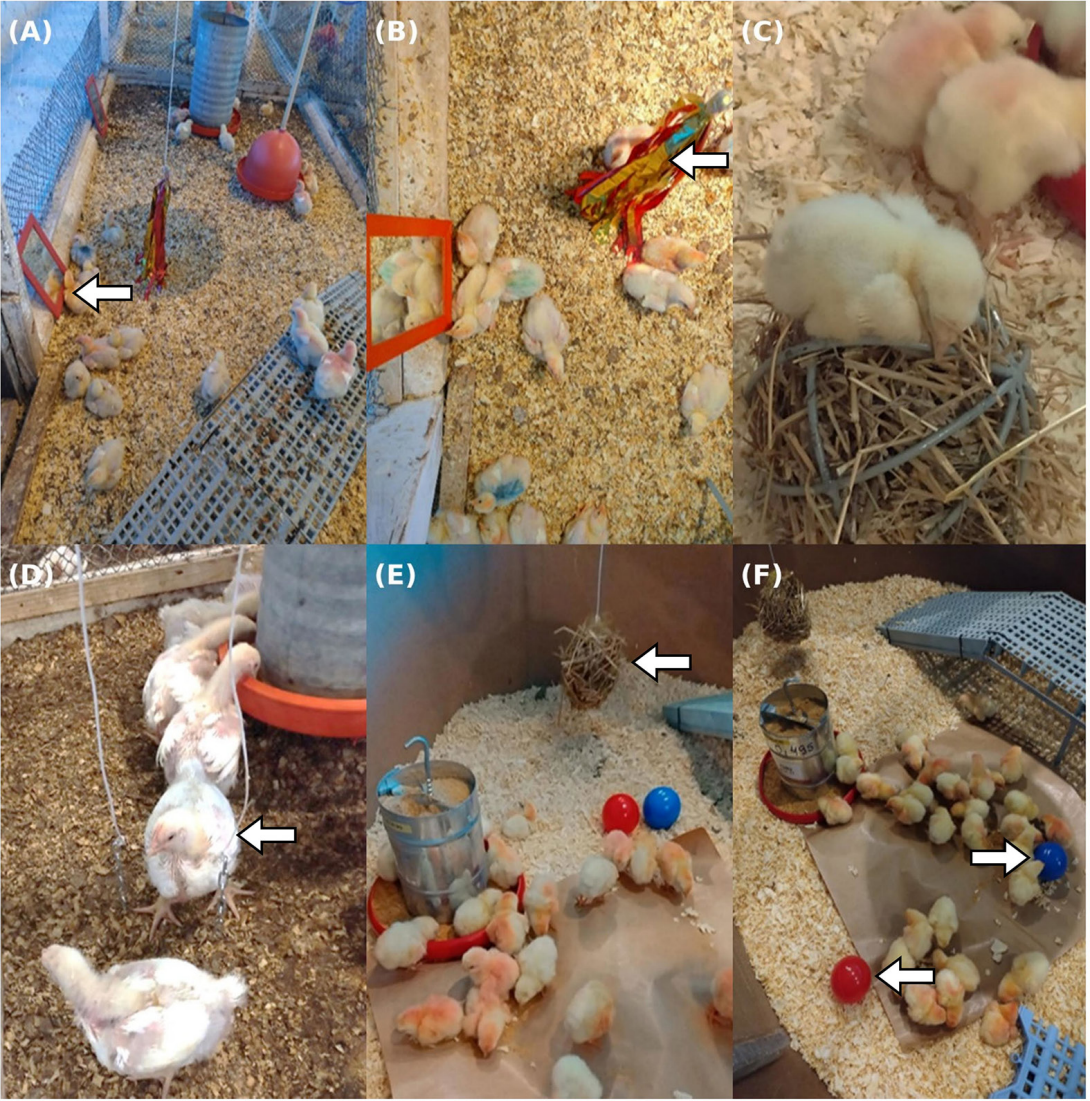
Temporary EE devices: (A) mirrors; (B) suspended metal strips; (C) alfalfa hay in perforated metal holders; (D) suspended iron chain; (E) suspended alfalfa hay; and (F) colored balls. *Source:* Authors.

Six temporary enrichment devices were rotated every 3 days among the enriched pens. Enrichment devices were rotated every 3 days to reduce habituation to individual objects and maintain birds' exploratory interest throughout the rearing period. This interval was selected as a practical management strategy rather than based on a predefined biological threshold. During each period, all enriched pens received the same enrichment item, while pens assigned to the non‐enriched treatment remained without enrichment. Group 1 consisted of mirrors positioned at bird height and suspended metal strips. Group 2 consisted of iron chains hanging at animal height, plus alfalfa hay provided in metal balls with openings (Figure 2).

### 2.2 Bone Densitometry

**FIGURE 3:**
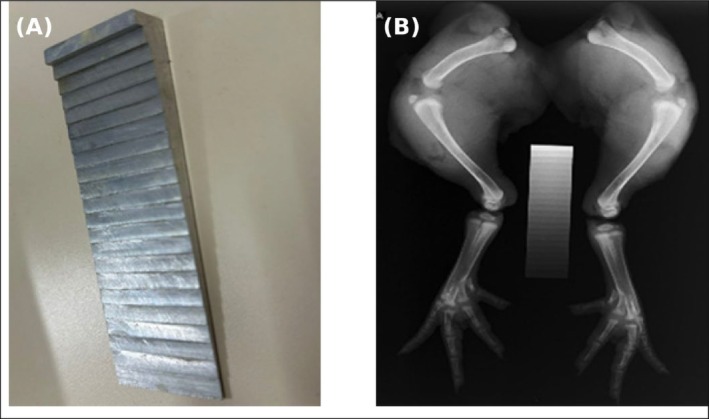
Radiographic bone densitometry of broiler chickens: (A) aluminum step wedge used for calibration; (B) radiographic image of the femur showing the evaluated regions. *Source:* Authors.

At 42 days of age, one bird per pen was selected and euthanized by cervical dislocation, following the approved animal ethics protocol for the collection of the femur and tibia, totaling 48 birds. The bones were radiographed together with an aluminum step wedge containing 22 steps, each ranging from 0.85 to 9.45 mm in thickness (Figure 3). Radiographs were obtained using an exposure setting of 47 kVp and 3 mAs, with a focal‐film distance of approximately 100 cm (JOB Corporation PORTA120HF‐Toshiba DF‐182). All radiographs were acquired under standardized geometric conditions, and no correction for radiographic magnification was applied. The aluminum step wedge included in each radiograph served as an internal calibration standard for bone densitometry. For regional evaluation, each bone was divided into three anatomical regions. In the femur, the regions corresponded to the proximal epiphysis (Region 1), diaphysis (Region 2), and distal epiphysis (Region 3). In the tibia, the regions corresponded to the proximal extremity (Region 1), diaphysis (Region 2), and distal extremity (Region 3). Radiographic bone densitometry was performed using ImageJ software, with bone mineral density values calibrated against the aluminum step wedge, and the results were expressed as millimeters of aluminum equivalent (mm Al).

### 2.3 Bone Strength

**FIGURE 4:**
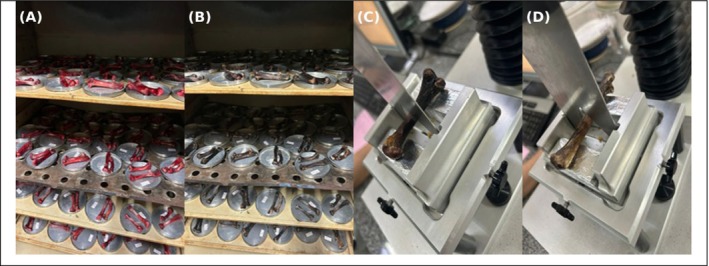
Procedures used for bone strength evaluation: (A) bone samples in a forced‐air oven; (B) samples after 24 h of drying; (C) femur during mechanical testing; and (D) tibia during mechanical testing. *Source:* Authors.

The left tibiae and femora were dried in a forced‐air oven at 105°C for 24 h and subsequently subjected to bone strength testing. Bone strength (kgf) was evaluated by a mechanical breaking test using a Texture Analyzer TA.XT Plus (Stable Micro Systems, UK) that is equipped with an HDP/BS Blade Set probe. The test was performed at a pre‐test speed of 4 mm/s and a displacement distance of 10 mm. The apparatus was adapted to provide a free span of 6.0 cm across the diaphysis, minimizing the influence of bone length on the measurements (Figure 4).

### 2.4 Tibial Dyschondroplasia

**FIGURE 5:**
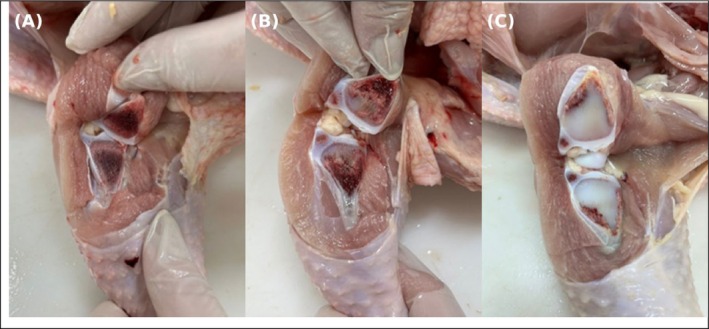
Representative scores used for TD assessment in broiler chickens: (A) normal growth plate; (B) intermediate cartilage thickening (1–3 mm); and (C) severe cartilage thickening (>3 mm). *Source:* Authors.

Tibial dyschondroplasia (TD) was evaluated by macroscopic examination of the proximal growth plate of both tibiae, following the methodology described by Alves et al. (2016). After longitudinal sectioning, samples were classified according to the degree of cartilage thickening as follows: score 0, absence of abnormal cartilage thickening; score 1, growth plate thickening between 1 and 3 mm; and score 2, growth plate thickening greater than 3 mm (Figure 5).

### 2.5 Spondylolisthesis

**FIGURE 6:**
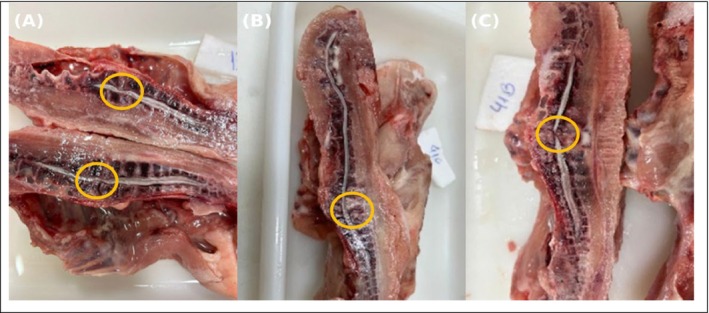
Representative macroscopic lesions of spondylolisthesis in broilers. Longitudinal sections of the vertebral column showing displacement of the thoracic vertebrae and consequent compression of the spinal cord (A–C). Samples were assigned a score of 1, indicating the presence of spondylolisthesis.

Spondylolisthesis was evaluated at 42 days of age using both macroscopic examination and computed tomography (CT). For macroscopic evaluation, three birds per pen were euthanized by cervical dislocation, following the approved animal ethics protocol, and their vertebral columns were removed, frozen at −10°C, and sagittally sectioned using a commercial band saw, as described by Mendonça Júnior (2009). The thoracic vertebrae between T4 and T7 were examined for evidence of spinal cord compression (Figure 6).

**FIGURE 7:**
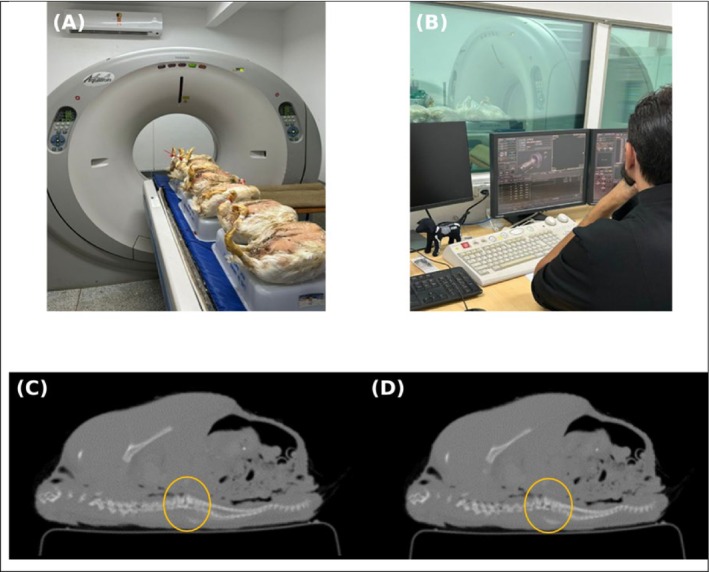
Evaluation of spondylolisthesis using CT scan (A), image of the CT scan procedure (B). Evaluation of spondylolisthesis with an image obtained from a CT scan, demonstrating the spinal cord. Letters C and D indicate the lesion.

For tomographic evaluation, one bird per pen (48 birds in total) was subjected to CT. The images were analyzed using RadAnt DICOM Viewer software, and the same vertebral region (T4–T7) was assessed for vertebral displacement and spinal cord compression (Figure 7).

For both evaluation methods, birds were classified as score 0 (absence of spondylolisthesis) when vertebrae were aligned normally without spinal cord compression, or score 1 (presence of spondylolisthesis) when vertebral displacement resulted in spinal cord compression.

### 2.6 Dorsocranial Myopathy

**FIGURE 8:**
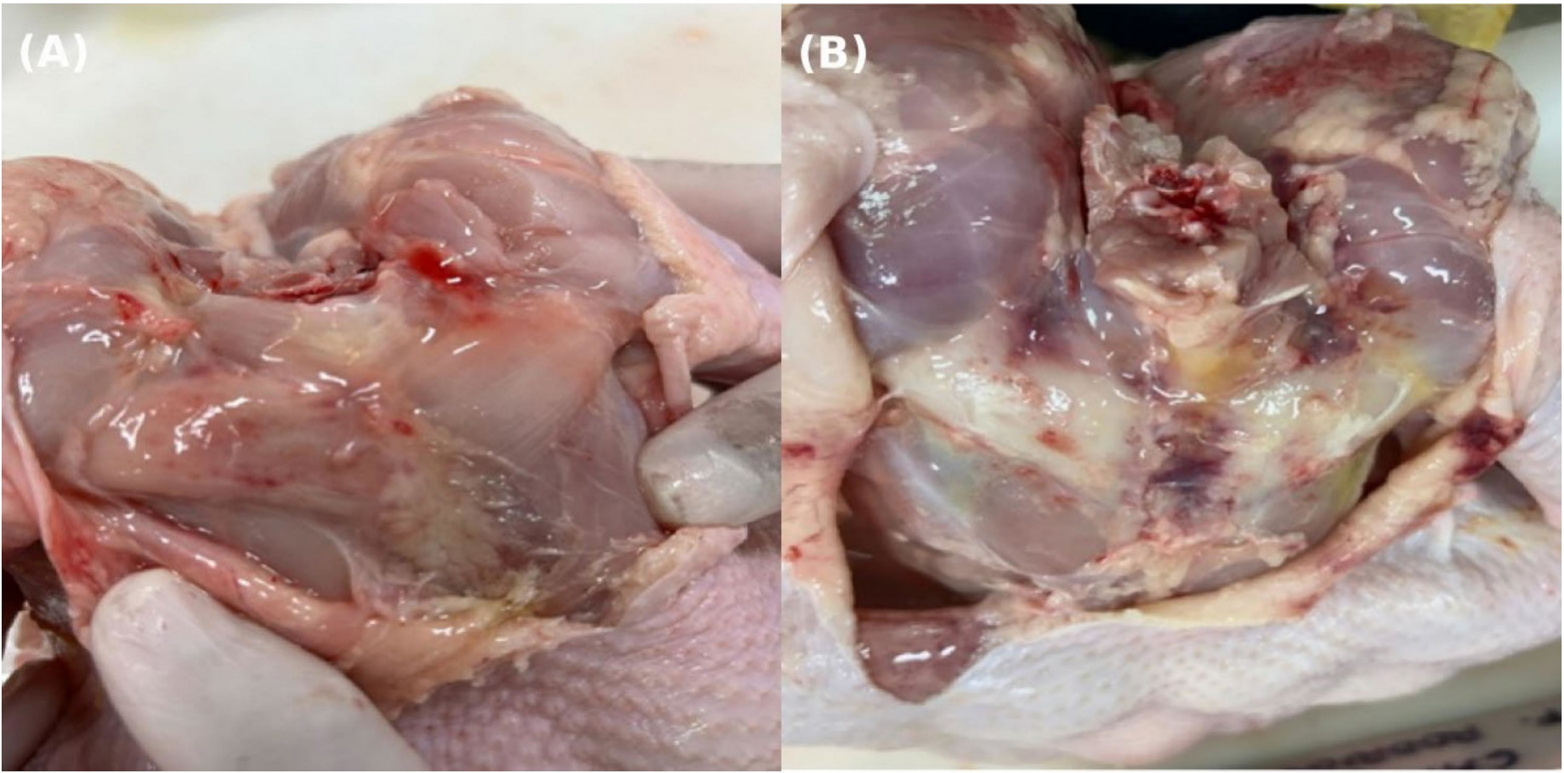
Macroscopic evaluation of DCM in broiler chickens: (A) score 2, moderate lesion characterized by yellowish discoloration and partial fibrosis; and (B) score 3, severe lesion characterized by extensive discoloration, necrosis, and tissue retraction. *Source:* Authors.

At 42 days of age, one bird per pen was euthanized by cervical dislocation, following the approved animal ethics protocol for the evaluation of dorsal cranial myopathy (DCM). After exposing the *M. latissimus dorsi pars cranialis* region, located between the base of the neck and the wing joint, lesions were visually assessed and classified according to severity as follows: score 0, absence of lesions; score 1, mild lesions characterized by small yellowish areas; score 2, moderate lesions with yellowish discoloration and partial fibrosis; and score 3, severe lesions characterized by extensive yellowing, necrosis, and tissue retraction (Figure 8).

### 2.7 Gait Score

From 23 days of age, the gait score was evaluated weekly following Dawkins et al. (2004). At each evaluation, birds were randomly selected from each experimental unit, and the same individuals were not followed throughout the experiment. Birds were classified as score 0 when able to walk 10 uninterrupted steps, score 1 when able to walk between 6 and 10 uninterrupted steps, and score 2 when unable to complete 6 uninterrupted steps.

### 2.8 Latency to Lie

Latency to lie (LTL) was evaluated according to the methodology described by Almeida Paz et al. (2010). The test was performed on 5% of the birds that were classified as gait score 0 and on all birds classified as gait score 1. Birds were placed in groups of three in a glass box (75 × 55 × 30 cm) containing 3 cm of room‐temperature water. The time elapsed until the first attempt to sit was recorded using a digital stopwatch. Birds that remained standing for 370 s were assigned the maximum test duration and removed from the evaluation.

### 2.9 Valgus and Varus

**FIGURE 9:**
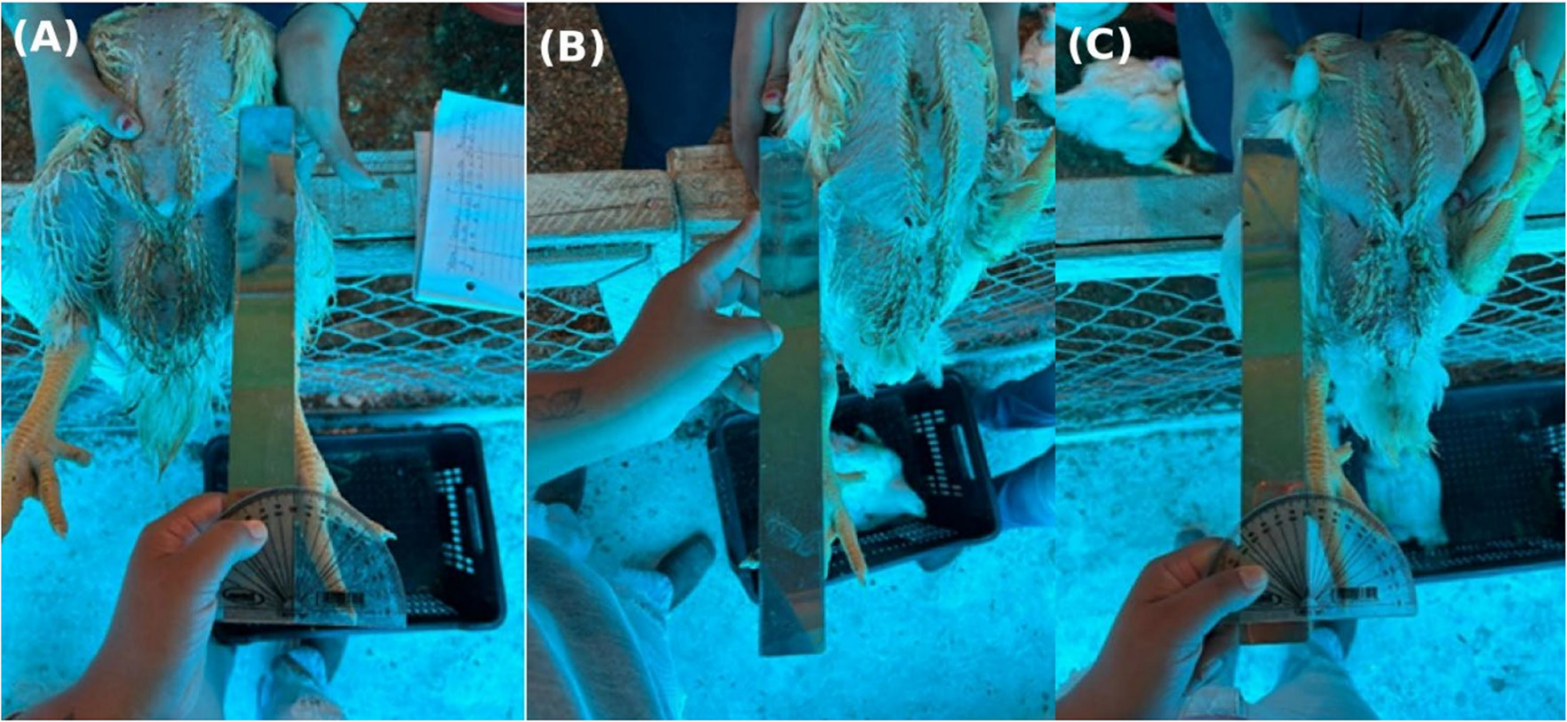
Representative angular limb conformations in broiler chickens: (A) valgus, (B) normal alignment, and (C) varus. *Source:* Authors.

Valgus and varus (V/V) deformities were assessed according to Almeida Paz et al. (2010) by measuring the angle between the longitudinal axis of the tibiotarsus and the third digit using a ruler and a protractor (Figure 9). Angular deviations directed laterally were classified as valgus, whereas deviations directed medially were classified as varus. Measurements were expressed in degrees.

### 2.10 Femoral Degeneration

**FIGURE 10:**
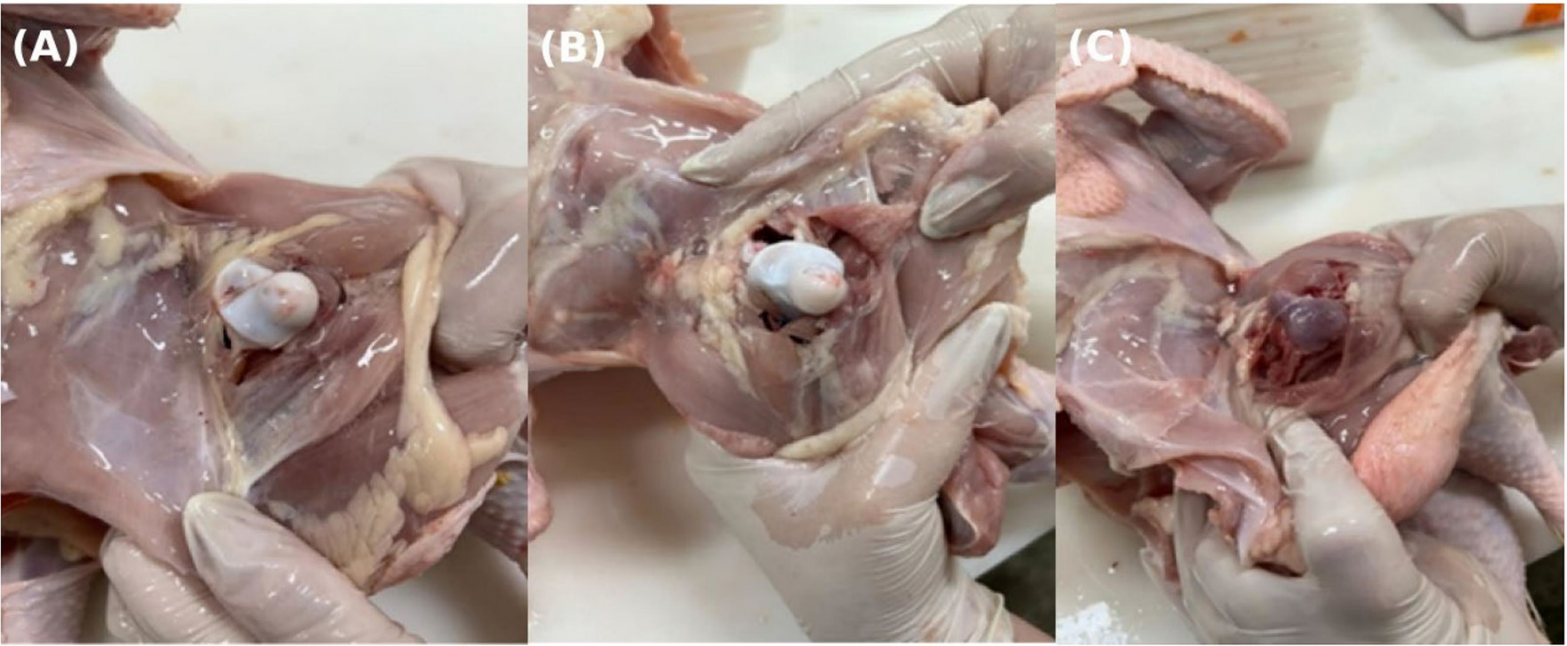
Macroscopic evaluation of FD in broiler chickens: (A) score 0, complete integrity of the articular cartilage; (B) score 1, partial loss of cartilage integrity; and (C) score 2, complete absence of articular cartilage. *Source:* Authors.

Femoral degeneration (FD) was evaluated by macroscopic examination of the femoral heads of both legs, following the methodology described by Alves et al. (2016). Samples were scored according to the integrity of the articular cartilage as follows: score 0, complete integrity of the femoral head; score 1, partial loss of cartilage integrity; and score 2, complete absence of articular cartilage (Figure 10).

### 2.11 Footpad Dermatitis

**FIGURE 11:**
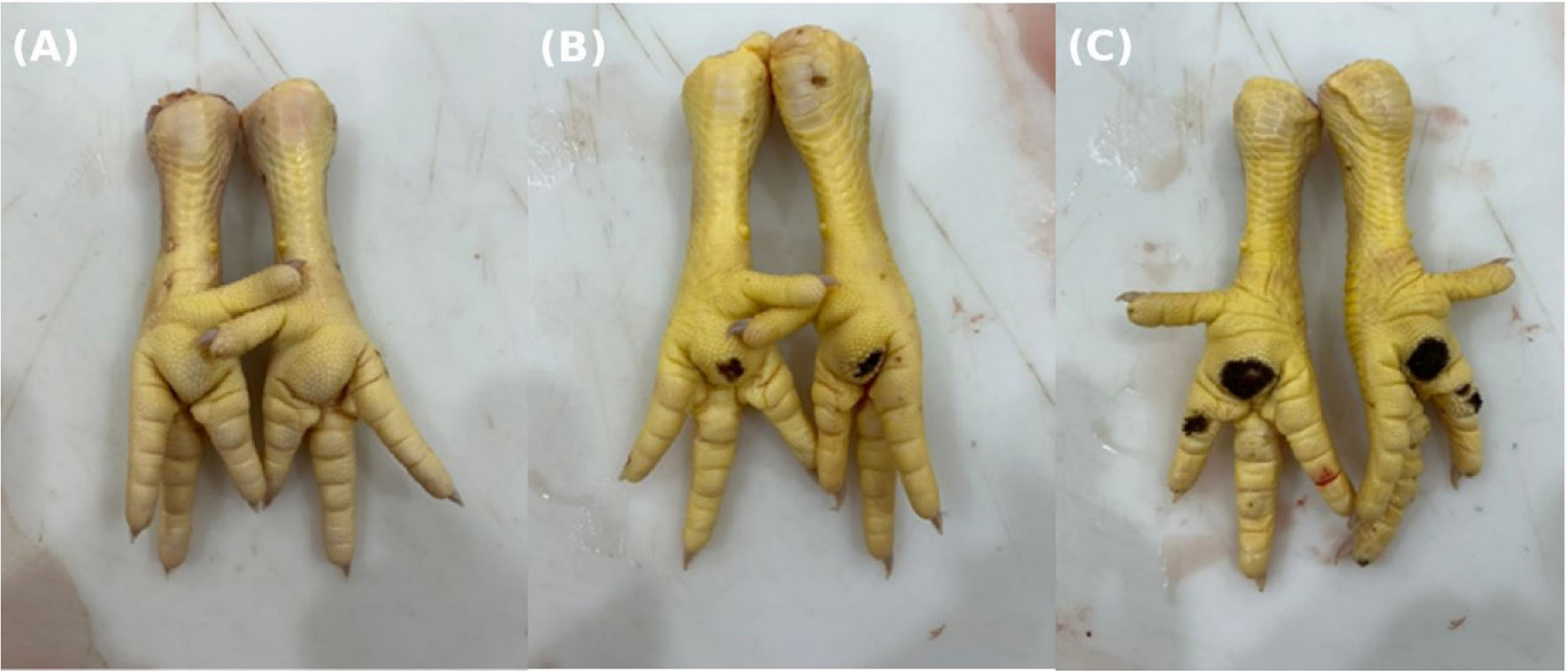
Representative scores for FPD in broiler chickens: (A) score 0, intact footpad without visible lesions; (B) score 1, mild lesion with a diameter of up to 5 mm; and (C) score 2, severe lesion with a diameter greater than 5 mm. *Source:* Authors.

At each evaluation age (days 23, 36, and 40), birds were assessed independently, and no individual identification was used to follow the same birds over time. Therefore, different birds could be evaluated at each sampling time. Footpad dermatitis (FPD) was assessed only in birds presenting a gait score greater than 0 at the respective evaluation. The plantar surface of the footpads was visually examined as described by Almeida Paz et al. (2010). Lesions were classified as follows: score 0, intact footpad without lesions; score 1, mild lesion with a diameter of up to 5 mm; and score 2, severe lesion with a diameter greater than 5 mm (Figure 11).

### 2.12 Statistical and Quantitative Analyses

The experimental unit was the pen (48 pens in total; 8 replicate pens per treatment). Individual birds evaluated within each pen were considered subsamples of the experimental unit.

LTL and bone densitometry data were evaluated for normality of residuals using the Shapiro–Wilk test and homogeneity of variances using Levene's test. Thus, an analysis of variance was performed using the PROC MIXED procedure. The statistical models included the type of additive used, EE, and their interactions. Replicate pen, nested within the treatment combination, was included as a random effect in all models to account for the correlation among birds housed within the same pen. For variables assessed on multiple birds within each pen (e.g., gait score, FPD, valgus/varus, and other locomotor traits), individual birds were treated as subsamples, whereas replicate pen was considered the experimental unit and included as the random effect in the mixed models to account for within‐pen correlation. At each assessment age, birds were randomly selected from each pen, and the same individuals were not repeatedly evaluated throughout the experiment. Therefore, observations obtained at different ages were considered independent rather than repeated measurements. When there were no interactions, the main effects means were compared using the same test.

The data obtained for locomotor and myopathy assessments were scores; therefore, they do not have a normal distribution. Thus, the theory of generalized linear models proposed by Nelder and Wedderburn (1972) was used, and the SAS GLIMMIX procedure was applied. The data distribution was LOGNORMAL, except for the spondylolisthesis data, which used a BINARY distribution. The LOGNORMAL distribution assumes that the logarithms of the data are normally distributed, while the BINARY distribution assumes that the residuals have Bernoulli/binomial distribution. The covariance structure in the GLIMMIX procedure was selected based on the corrected Akaike Information Criterion (AICC). Among the competing models, the covariance structure with the lowest AICC value was selected because this criterion provides a more reliable model comparison in finite samples by accounting for model complexity.

The statistical models included the type of additive used, EE, and their interactions. The same fixed and random effects were used in the generalized linear mixed models. When the right and left sides of the animals' paws were evaluated separately, the side effect was modeled as a random effect (RANDOM command). Generalized linear mixed models were fitted using the default estimation method implemented in PROC GLIMMIX (SAS Institute Inc., Cary, NC, USA).

To compare the means using the “lsmeans” test, the “estimates” obtained were used in “inverse link” functions and adjustments (pdiff ilink lines) of the GLIMMIX procedure. Least squares means were back‐transformed model estimates to the original response scale. Therefore, all means presented in the tables correspond to back‐transformed values. When applicable, the corresponding standard errors and confidence intervals were also back‐transformed to the original response scale.

The variable valgus/varus is presented as the average deviation from the 0° angle, independent of direction; that is, how much each limb of the bird deviates on average from the 0° angle. The higher the value, the greater the deviation from the 0° angle, and the more severe the lesion. The variables pododermatitis and gait score were evaluated using estimates from the chosen models but are presented in the tables as averages for ease of understanding; higher values indicate a greater degree of lesion. The variable spondylolisthesis, being a binary data point, is expressed, for ease of understanding, as the number of animals with and without the alteration, as a function of the number of animals evaluated in the experimental group.

## 3 Results

### 3.1 Bone‐Related Parameters

No significant interaction between additives and EE was observed for femoral or tibial bone density (*p* > 0.05). Likewise, dietary additives did not affect bone density (*p* > 0.05). However, birds reared in enriched environments exhibited higher bone density values in both the femur and tibia than those reared without enrichment (*p* < 0.05). Differences were observed among anatomical regions of both bones (*p* < 0.05). In the femur, the highest density values were recorded in the diaphyseal and distal regions (Regions 2 and 3), whereas in the tibia, the proximal region (Region 1) showed the greatest density (Table 2).

**TABLE 2:** Bone mineral density of the femur and tibia in broiler chickens fed different dietary additives and reared with or without EE.

Bone	Region	With EE			Without EE			Region mean
		AGP	CN	PROB	AGP	CN	PROB	
Femur	1	4.98	4.92	5.14	4.85	4.79	4.63	4.88B
	2	6.21	5.99	6.38	5.89	6.05	6.11	6.11A
	3	6.29	6.27	6.32	6.04	6.06	6.35	6.22A
	Mean EE	5.84a			5.64b			5.73
Tibia	1	6.10	6.42	6.40	5.90	6.14	6.30	6.21A
	2	5.42	5.43	5.28	5.20	5.05	5.35	5.29B
	3	4.04	4.15	3.95	3.96	4.15	4.09	4.02C
	Mean EE	5.24a			5.10b			5.18
	SEM and probability values							
	SEM	Additive	EE	Region	A × EE	A × R	EE × R	A × EE × R
Femur	0.123	0.2607	0.0058	<0.0001	0.6128	0.8286	0.8208	0.3890
Tibia	0.110	0.2651	0.0321	<0.0001	0.1154	0.2405	0.6118	0.9277

*Note:* Bone density was evaluated in three anatomical regions of each bone: Region 1 (proximal epiphysis/proximal extremity), Region 2 (diaphysis), and Region 3 (distal epiphysis/distal extremity), and the results were expressed as millimeters of aluminum equivalent (mm Al).

Abbreviations: AGP, antibiotic growth promoter; CN, negative control; EE, environmental enrichment; PROB, probiotic; SEM, standard error of the mean. Myopathy means followed by different uppercase letters within a column and different lowercase letters within a row differ significantly according to Tukey's test (*P* < 0.05).

Across anatomical regions, differences were observed among regions (*p* < 0.05), with higher density values recorded in the diaphyseal and distal regions of the femur (Regions 2 and 3) and in the proximal region of the tibia (Region 1). These results suggest region‐specific bone adaptation, likely associated with differential mechanical loading.

Despite the differences observed in bone density, no significant effects of dietary additives, EE, or their interaction were detected on femoral or tibial bone strength (Table 3).

**TABLE 3:** Femoral and tibial bone strength (kgf) of broiler chickens fed different dietary additives and reared with or without EE.

Bone	Additive	EE			SEM	Probabilities		
		With EE	Without EE	Mean		Additive	EE	A × EE
Femur	AGP	31.57	32.75	32.16	1.14	0.2408	0.7631	0.9403
	CN	37.05	36.61	36.83				
	PROB	34.69	36.02	35.36				
	Mean	34.44	35.13	34.78				
Tibia	AGP	31.79	35.59	33.69	1.13	0.4241	0.6302	0.6354
	CN	31.98	30.37	31.18				
	PROB	34.24	35.41	34.83				
	Mean	32.67	33.79	33.23				

Abbreviations: AGP, antibiotic growth promoter; CN, negative control; EE, environmental enrichment; PROB, probiotic; SEM, standard error of the mean.

### 3.2 Cartilage and Structural Disorders

A significant additive EE interaction was observed for FD (Table 4; *p* < 0.05). Under enriched conditions, birds receiving probiotics exhibited lower FD scores than those receiving an antibiotic growth promoter or the control diet, whereas no differences among dietary treatments were observed in the absence of EE. In contrast, no significant additive EE interactions were detected for TD or DCM. Likewise, neither dietary additives nor EE affected TD or DCM when evaluated separately.

**TABLE 4:** TD, FD, and DCM scores in broiler chickens fed different dietary additives and reared with or without EE.

Lesion	Additive	EE			SEM	Probabilities		
		With EE	Without EE	Mean		Additive	EE	A × EE
TD	AGP	0.210	0.040	0.130	0.05	0.1375	0.5387	0.1614
	CN	0.350	0.170	0.260				
	PROB	0.160	0.150	0.150				
	Mean	0.240	0.120	0.180				
FD	AGP	0.682Aa	0.515Aa	0.599	0.20	0.8411	0.1036	0.0463
	CN	0.562Aa	0.649Aa	0.606				
	PROB	0.409Ba	0.411Ba	0.410				
	Mean	0.551	0.524	0.540				
DCM	AGP	0.391	0.391	0.391	0.10	0.1775	0.1655	0.1785
	CN	0.260	0.260	0.260				
	PROB	0.272	0.291	0.282				
	Mean	0.308	0.314	0.311				

Abbreviations: AGP, antibiotic growth promoter; CN, negative control; DCM, dorsal cranial myopathy; EE, environmental enrichment; FD, femoral degeneration; PROB, probiotic; SEM, standard error of the mean; TD, tibial dyschondroplasia. Means followed by different uppercase letters within a column and different lowercase letters within a row differ significantly according to Tukey's test (*P* < 0.05).

Spondylolisthesis, assessed by both CT and visual inspection, was not influenced by dietary additives, EE, or their interaction (Table 5). The incidence of the condition was low across all treatment groups; thus, the statistical power to detect treatment differences was limited.

**TABLE 5:** Incidence of spondylolisthesis in broiler chickens fed different dietary additives and reared with or without EE, assessed by CT and visual inspection.

Spondylolisthesis	Additive	With EE		Without EE		Probabilities		
		+	−	+	−	Additive	EE	A × EE
CT	AGP CN	4.2%	95.8%	6.2%	93.8%	0.9994	0.9986	0.9993
		0%	100%	8.3%	91.7%			
	PROB	6.2%	93.8%	0%	100%			
Visual	AGP CN	12.5%	87.5%	16.6%	83.4%	0.2051	0.1186	0.2354
		20.8%	79.2%	4.2%	95.8%			
	PROB	33.3%	66.7%	16.6%	83.4%			

Abbreviations: AGP, antibiotic growth promoter; CN, negative control; CT, computed tomography; EE, environmental enrichment; PROB, probiotic. *p*‐values were obtained from generalized linear mixed models assuming a binary distribution (PROC GLIMMIX).

### 3.3 Locomotor Performance and Posture‐Related Variables

LTL and valgus/varus angular deviations (V/V) were not affected by the additive × EE interaction, nor by the main effects of dietary additives and EE (Table 6).

**TABLE 6:** LTL valgus/varus angular deviations and gait score in broiler chickens fed different dietary additives and reared with or without EE.

Lesion	Additive	EE			SEM	Probabilities		
		With EE	Without EE	Mean		Additive	EE	A × EE
LTL (seg)	AGP	94.45	107.46	100.96	1.96	0.4792	0.7703	0.6665
	CN	121.75	117.12	119.94				
	PROB	108.38	85.95	97.16				
	Mean	100.19	103.51	106.02				
V/V	AGP	5.00	4.58	4.79	1.17	0.8665	0.1673	0.0554
	CN	7.71	3.13	5.41				
	PROB	4.58	5.62	5.10				
	Mean	5.76	4.44	5.10				
Gait score day 23	AGP	0.041Ba	0.083Aa	0.062	0.06	0.0007	0.1679	0.0007
	CN	0.166Aa	0.083Aa	0.126				
	PROB	0.083ABa	0.166Aa	0.126				
	Mean	0.097	0.111	0.104				
Gait score day 36	AGP	0.000	0.217	0.108B	0.07	<0.0001	<0.0001	0.1869
	CN	0.130	0.170	0.150A				
	PROB	0.130	0.170	0.145A				
	Mean	0.086b	0.185a	0.136				
Gait score day 40	AGP	0.220	0.430	0.325	0.11	0.0840	0.2015	0.1411
	CN	0.380	0.380	0.380				
	PROB	0.500	0.480	0.490				
	Mean	0.343	0.430	0.386				

Abbreviations: AGP, antibiotic growth promoter; CN, negative control; EE, environmental enrichment; LTL, latency to lie; PROB, probiotic; SEM, standard error of the mean; V/V, valgus and varus myopathy. Means followed by different uppercase letters within a column and different lowercase letters within a row differ significantly according to Tukey's test (*P* < 0.05).

Gait score at 23 days of age was influenced by the interaction between dietary additives and EE. Under enriched conditions, birds receiving the antibiotic growth promoter exhibited lower gait scores than those fed the control diet, whereas probiotic‐fed birds showed intermediate values. In the absence of EE, no differences among dietary treatments were observed.

At 36 days of age, no interaction between additives and EE was detected. However, significant main effects were observed for both factors. Birds receiving antibiotic growth promoter exhibited lower gait scores than those receiving the probiotics or the control diet. In addition, birds reared in enriched pens showed lower gait scores than those reared without EE.

### 3.4 FPD

FPD severity increased with age. No additive EE interaction was observed at any evaluation age, and EE did not affect lesion scores. On 23 days, no differences among dietary treatments were detected. At 36 days, birds receiving the antibiotic growth promoter showed the lowest pododermatitis scores, followed by probiotic‐fed birds. At 40 days, both dietary additives reduced lesion severity compared with the control treatment (Table 7).

**TABLE 7:** FPD severity scores in broiler chickens fed different dietary additives and reared with or without EE at 23, 36, and 40 days of age.

Lesion	Additive	EE			SEM	Probabilities		
		With EE	Without EE	Mean		Additive	EE	A × EE
Day 23	AGP	0.63	0.58	0.60	0.07	0.3178	0.0874	0.681
	CN	0.79	0.63	0.71				
	PROB	0.67	0.46	0.56				
	Mean	0.69	0.56	0.62				
Day 36	AGP	0.71	0.70	0.702b	0.09	0.0405	0.5121	0.9176
	CN	0.96	0.88	0.916a				
	PROB	0.79	0.75	0.770ab				
	Mean	0.82	0.77	0.80				
Day 40	AGP	0.74	0.780	0.760b	0.07	0.0009	0.2238	0.8046
	CN	1.00	1.040	1.02a				
	PROB	0.79	0.910	0.852b				
	Mean	0.84	0.91	0.88				

Abbreviations: AGP, antibiotic growth promoter; CN, negative control; EE, environmental enrichment. LTL, latency to lie; PROB, probiotic; SEM, standard error of the mean; V/V, valgus and varus. Means followed by different lowercase letters within the same bone differ significantly (*p* < 0.05).

## 4 Discussion

The present study was based on the hypothesis that combining dietary additives with EE would synergistically improve the locomotor health of broiler chickens. This hypothesis was only partially supported by the results. Significant interactions between these factors were observed only for FD and gait score at 23 days, whereas most locomotor traits were affected independently by dietary additives or EE. These findings suggest that, although the combination of nutritional and environmental strategies may provide complementary benefits under specific conditions, their effects were generally additive or independent rather than synergistic.

EE positively affected femoral and tibial bone densitometry, indicating that greater environmental complexity may favor skeletal mineralization in broilers. This response is biologically plausible, as ramps and manipulable objects can stimulate exploratory behavior, walking, climbing, and postural adjustments, thereby increasing loading on the appendicular skeleton. Bone tissue responds dynamically to such stimuli through remodeling processes, which may explain the higher density values observed in enriched environments. Similar findings have been reported in broilers exposed to EE, particularly when structures such as perches, ramps, or elevated platforms promoted greater activity and altered locomotor behavior (Riber et al. 2018; Nazareno et al. 2022; Nazareno et al. 2024).

Despite the increase in bone density, no corresponding improvement in bone strength was observed. This suggests that enhanced mineral deposition alone was insufficient to modify the mechanical properties of the bones under the conditions of the present study. Bone strength is determined not only by mineral density but also by factors such as bone geometry, collagen matrix organization, and cortical architecture, which may explain the absence of treatment effects on this parameter (Fleming 2008; Yair et al. 2015).

Another factor that may have contributed to the lack of differences in bone strength is the mechanical testing of dried bones. Dehydration alters the mechanical properties of bone by reducing its water content, increasing stiffness and brittleness, and modifying the contribution of the collagen matrix to energy absorption during loading. Consequently, the use of dried specimens may have reduced the sensitivity of the mechanical test to detect subtle treatment‐related differences in bone strength, despite the observed differences in bone mineral density (Currey 2002).

TD was not affected by dietary additives or EE, reinforcing the multifactorial nature of this disorder. TD is associated with alterations in cartilage maturation, vascularization, mineralization, and endochondral ossification, processes that are strongly influenced by genetic growth potential, metabolic status, and nutritional balance (Kulyar et al. 2022; Shi et al. 2024). Consequently, management interventions such as EE or dietary supplementation with performance‐enhancing additives may be insufficient, by themselves, to modify the incidence of this condition.

In contrast, FD was influenced by the interaction between additives and EE, with lower lesion scores observed in probiotic‐fed birds under enriched conditions. This finding suggests a potential synergistic effect between improved intestinal function and increased physical activity in maintaining femoral cartilage integrity. Probiotics may enhance mineral utilization, modulate inflammatory responses, and influence the gut–bone axis, a pathway increasingly recognized as an important regulator of skeletal development (Markowiak and Śliżewska 2018; Mohammed et al. 2020, 2021; Tu et al. 2021). In addition, microbial metabolites such as butyrate have been implicated in bone remodeling and may influence chondrocyte function through immunomodulatory pathways, providing a biologically plausible link between intestinal health and cartilage integrity (Lucas et al. 2018; Zaiss et al. 2019). However, because neither gut microbiota composition nor markers of mineral metabolism were evaluated in the present study, these proposed mechanisms remain speculative and require further mechanistic validation.

Spondylolisthesis had a low incidence and was unaffected by dietary additives or EE, regardless of whether assessed by visual inspection or CT. These findings suggest that the evaluated interventions were insufficient to modify the risk of this vertebral disorder during the production cycle. Spondylolisthesis is a complex skeletal condition associated with rapid growth, vertebral instability, and alterations in axial skeletal development, and its occurrence is largely influenced by genetic and biomechanical factors that may not be readily altered by isolated nutritional or environmental strategies (Riddell 1973; Dinev 2014; Liu et al. 2023).

The use of CT offers a methodological advantage by enabling a more detailed assessment of vertebral structures. However, the low incidence of affected birds likely reduced the ability to detect treatment‐related differences. Similarly, DCM was not influenced by dietary additives or EE. This result supports the hypothesis that the condition is more closely associated with rapid growth, muscle hypertrophy, and biomechanical imbalance than with the management factors evaluated in the present study (Pavanello et al. 2022; Silva et al. 2025).

LTL and valgus/varus angular deviations were not affected by dietary additives or EE. These traits likely require more pronounced or sustained changes in locomotor activity to produce measurable responses. Although EE may stimulate movement, the frequency and intensity of birds' interactions with enrichment devices may vary considerably and may not be sufficient to influence postural characteristics or limb angulation. Previous studies have reported inconsistent effects of EE on locomotor welfare, with responses depending on factors such as the type, accessibility, and attractiveness of the enrichment, as well as stocking density, age, and genotype (Sans et al. 2014; Tahamtani et al. 2019; Weimer et al. 2020).

Therefore, the absence of significant effects on LTL and angular deformities should not necessarily be interpreted as evidence that enrichment lacks biological relevance; it suggests that these indicators were less responsive to the nutritional and environmental interventions evaluated in the present study.

A limitation of this study is that the birds selected for the LTL test were not sampled in proportion to the flock distribution of gait scores. Instead, all birds with gait score 1 and only a random subset of birds with gait score 0 were evaluated to ensure adequate representation of individuals with mild locomotor impairment. Consequently, the LTL results should be interpreted as a comparison among experimental treatments within the selected sample rather than as an estimate of locomotor performance or impairment prevalence for the entire flock.

Gait score responses were age‐dependent; thus, at 23 days of age, the interaction between dietary additives and EE indicated that birds receiving the antibiotic growth promoter under enriched conditions exhibited the lowest gait scores, while probiotic‐fed birds showed intermediate values. At 36 days, the antibiotic growth promoter resulted in the most favorable gait scores, whereas by 40 days, no treatment differences were detected. This temporal pattern suggests that nutritional and environmental interventions may exert greater influence during the intermediate stages of growth, before the effects of increased body weight and age‐related locomotor constraints become more pronounced. The beneficial effects observed in probiotic‐fed birds may be related to improvements in gut health, nutrient utilization, and immune regulation. In addition, probiotics may contribute to better litter quality by influencing excreta characteristics, thereby indirectly supporting locomotor welfare (Santini et al. 2010; Rehman et al. 2020; Hu et al. 2022). However, the absence of treatment effects at 40 days suggests that these benefits may become less evident as birds approach market age, when the rapid increase in body mass and the associated mechanical loading on the skeletal system may outweigh the nutritional advantages provided by probiotics. Reduced activity and the cumulative effects of growth‐related locomotor stress may further limit the expression of these benefits (Knowles et al. 2008; Liu et al. 2023).

FPD increased with age, probably due to prolonged exposure of the footpad to litter moisture and excreta, and to the greater pressure imposed by increasing body weight. At 36 and 40 days, birds receiving antibiotics or probiotics had lower lesion scores than the control group, suggesting that dietary modulation improved footpad health. This effect may be related to improvements in intestinal function, nutrient utilization, and litter quality, all of which are recognized factors associated with the development of pododermatitis (Shepherd and Fairchild 2010; Taira et al. 2013; Durmus et al. 2023). However, litter moisture and other indicators of litter quality were not measured in the present study. Therefore, the proposed relationship between dietary additives, litter conditions, and reduced pododermatitis remains speculative and should be interpreted with caution. EE did not consistently affect lesion scores, indicating that under the conditions of the present study, dietary effects on intestinal and litter‐related factors were more important than enrichment‐induced behavioral changes.

Overall, the results indicate that EE and dietary additives affected different aspects of locomotor health in broiler chickens. EE primarily enhanced bone mineralization, whereas dietary additives, particularly probiotics, were associated with improvements in selected welfare indicators, including FD, gait score, and pododermatitis. These findings suggest that nutritional and environmental strategies may act through complementary pathways, with enrichment promoting skeletal development and dietary additives contributing more directly to locomotor performance and footpad health.

The results should not be interpreted as evidence of equivalence between probiotics and antibiotic growth promoters, since formal equivalence analyses were not performed. Furthermore, only one commercial broiler genetic line (Ross TM4) was evaluated. Therefore, caution should be exercised when extrapolating these findings to other broiler genotypes, as responses to EE and dietary additives may vary according to genetic background, growth rate, and skeletal conformation.

Another limitation of the present study is that the use of the EE devices was not quantified through behavioral observations. Although the enrichments were continuously available to the birds, the frequency and duration of their use were not recorded. Consequently, it was not possible to directly relate the observed locomotor outcomes to increased physical activity or interaction with the enrichment devices.

In addition, variables potentially involved in the proposed mechanisms, such as gut microbiota composition, intestinal morphology, litter quality, and metabolomic responses, were not directly measured. Likewise, the low incidence of some disorders, particularly spondylolisthesis, may have reduced the statistical power to detect treatment effects.

These limitations should be considered when interpreting the present findings but do not diminish the observed beneficial effects of probiotics and EE on several indicators of locomotor health. Future studies integrating behavioral assessments of enrichment use, microbiota profiling, litter quality evaluation, metabolomic analyses, and formal non‐inferiority or equivalence approaches across different commercial broiler genotypes will help clarify the mechanisms underlying these responses and better establish the potential of probiotics as alternatives to antibiotic growth promoters.

## 5 Conclusion

EE increased femoral and tibial bone mineralization but had limited effects on most locomotor and skeletal disorders evaluated. Dietary additives exerted a greater influence on welfare‐related indicators, with probiotics improving FD, gait score, and pododermatitis. These findings indicate that EE and dietary additives affect distinct aspects of locomotor health in broiler chickens, with probiotics emerging as a promising strategy to support locomotor welfare.

## Conflicts of Interest

The authors declare no conflicts of interest.

## Disclosing Artificial Intelligence Use

The authors used ChatGPT (OpenAI) to assist with language editing, grammar correction, and text organization. The authors reviewed and edited the generated content as necessary and take full responsibility for the content of the publication.

## Data Availability

The data that support the findings of this study are available from the corresponding author upon reasonable request.
